# Diagnostic Pathology in 2010: the successes and perspectives of open access publication

**DOI:** 10.1186/1746-1596-6-2

**Published:** 2011-01-07

**Authors:** Klaus Kayser

**Affiliations:** 1 Director of the UICC-TPCC, Humboldt University (Charite), Institute of Pathology, Berlin, Germany

## 

Electronic information transfer has replaced previous written information in nearly, if not all fields of communication to a high percentage. This method includes social information which is commonly spread by radio and television (and only delayed and less intensively distributed by newspapers), as well as all fields of science. This information can be classified into "spread of research results and ideas" (scientific journals) and into "direct research participation" (clinical trials, high energy experiments, etc.). Open access publication is a specific form of electronic information transfer. It is based upon an open information transfer medium such as the internet, and offers a non-commercial, i.e., free, access to read all included articles. The payment for this is provided by the authors (or their institutions), and interested companies by advertisement. Obviously, open access can be easily provided in electronic communication, and is, in general, difficult to be implemented by non-electronic media worldwide.

Our open access journal *Diagnostic Pathology *can be considered as a good example for the advantages and disadvantages of open access publication in surgical pathology (tissue - based diagnosis) and related fields. It started as a completely new journal (without any pre-existing conventional or electronic journal) in March 2006. From the beginning it was created as an independent scientific journal. The authors keep their rights, BioMed Central (the publisher) regulates the financial aspects of the journal and its layout, the Editor and the Editorial board are responsible for the scientific quality of the published articles, and the staff at BioMed Central is responsible for formal correctness and quality.

Disadvantages of open access publication have been mentioned as non-negligible financial burden to the authors, missing scientific quality because outstanding scientists and researchers prefer to publish in "conventional structured journals", easy, non-controlled "black" copies of compartments of or complete published articles, and new, often hard to evaluate mixture of articles that focus on quite different medical (or scientific) fields, i.e. that are completely organized in a "databank environment".

What are the experiences with our journal *Diagnostic Pathology *in the last five years? We have to admit, that, at the beginning in 2006, we had great doubts about the willingness of authors and their institutions to invest their money in electronic publication, and in a "fresh" journal without any reputation, as it was not yet listed in the Thomson Reuter's Citation Index. It needed about six months to overcome this constraint, and after one year it seemed that the journal had found a solid basis of existence. One reason can be found in the acceptance of case reports, which was the focus of the majority of published articles then. Quite often these articles were submitted from colleagues working in developing countries and we were glad to assist them in publication. On the other hand, we had our doubts upon this kind of publication, as these case reports might depress or even block the evaluation and acceptance of the journal by Thomson Reuter's Citation Index.

In addition, we always took care that our colleagues, namely pathologists, could read articles they are interested in, in a conventional manner. This is assured by the formal appearance of the journal on its homepage, with only minor disturbances from advertisement or subjects that are related to other medical fields.

The advantages of open access publication include fast and world-wide spread of the published articles, no costs for the readers, control of access to specific articles (number of hits), followed by article ranking and evaluation of scientific interest of the readers, fast publication, and potential implementation of future publication features.

The recent development of our journal *Diagnostic Pathology *might be useful for comparing the disadvantages with the advantages. A milestone in the journal's progress was the inclusion into the citation index carousel in 2010. A citation index of 1.3 has been received despite all fears that case reports might block this attribute. We have still to work on increasing the citation index, because it is the major justification to receiving money for publication from various resources. However, our limitation is that we still want to assist colleagues from developing countries, and to encourage them in publication in our journal.

The number of submitted and published articles has increased after the inclusion into the citation index library by nearly double within the last year. The percentage of submitted articles which we had to reject remained constant at about 35%. Thus, the citation index is one major component to secure the submission of articles.

In aggregate, to assure certain constancy in the scientific quality, the kind of accepted articles and official reputation are significant conditions to successfully establish and run an open access journal such as *Diagnostic Pathology*.

What are the perspectives of our journal? When analyzing electronic open access publication, potential new developments, the most of our published articles contain histological images, most frequently derived from immunohistochemically stained slides. These slides display certain specific areas, which are thought to be representative for the disease under discussion. In close collaboration with companies in the scanning industry (Leica Microsystems, DiagnomX) we will be able to publish not only specific areas (regions of interest, ROI), but, in addition, completely digitized slides which contain the ROIs.

**Figure 1:**
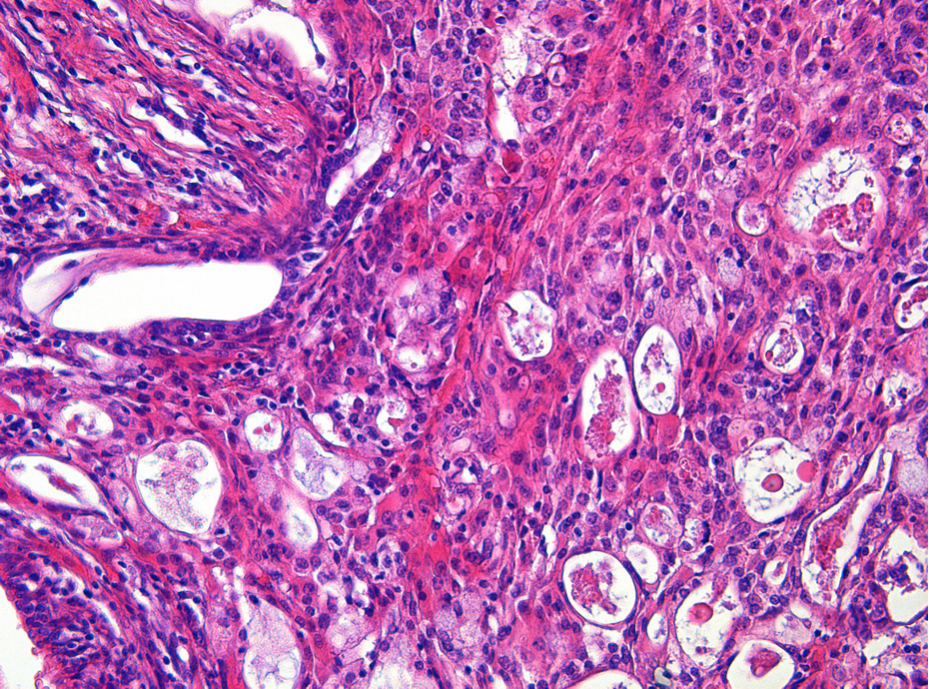
Microphotograph of the region of interest (ROI) of a mucoepidermoid carcinoma of the lung (HE, *200). To view the virtual slide for this image please see here http://eu-demo-dih.slidepath.com/dih/webViewer.php?snapshotId=1294305419

An example of this technique is shown in Figure 1. It displays a mucoepidermoid carcinoma of the lung which corresponds to the ROI of the underlying digitized complete histological slide (virtual slide).

The reader can verify the correct selection (in this case automatically computed) of the ROI by navigating through the whole digitized slide. The viewer and the virtual slide will be uploaded just by clicking on the image.

Navigation through the whole image, magnification of any areas at all microscopic levels as well as photographic shots are possible.

Starting in 2011, the authors of any accepted article are free to select this advanced technology at no additional cost, or can alternatively choose to stay with the conventional method of displaying images.

All in all, the year 2010 was a successful year. Having received the Impact Factor, both the number of submissions as well as the scientific quality of these manuscripts increased. We are now in the situation to include more advanced technologies in our journal. The readers' access to virtual slides will be the first step, and others will follow based upon the collected experiences. The final aim will be to use all appropriate items which are offered by electronic media to distribute and further analyze scientific information submitted for publication in our journal *Diagnostic Pathology*.

The authors, interested readers, the Editorial board and BioMed Central team remain, of course, the sound basis in running any kind of peer reviewed scientific journal. Certainly, we will not forget their significant influence and contribution to the obtained success and further development of our journal. We appreciate very much all their efforts and identification with our journal.

We want to express our deep gratitude to, and we wish all our authors, readers, reviewers, and our publication team a Merry Christmas and a Happy and Healthy New Year.

Klaus Kayser

Editor in Chief

